# Efflux-Mediated Macrolide Resistance in Clinical *Streptococcus* Isolates: A Comparative Molecular Study

**DOI:** 10.3390/antibiotics14111148

**Published:** 2025-11-13

**Authors:** Salsabeel M. Moshewh, Salma E. Mohamed, Praveen Kumar, Abdelgadir E. Eltom, Supriya R. Jagdale, Einas A. Osman, Saher S. Ahmed, Nour A. M. Farajallah, Sara Ali

**Affiliations:** 1 Department of Medical Laboratory Sciences, College of Health Sciences, Gulf Medical University, Ajman P.O. Box 4184, United Arab Emirates; 2023mscmls08@mygmu.ac.ae (S.M.M.); dr.abdel@gmu.ac.ae (A.E.E.); nour.farajallah7@gmail.com (N.A.M.F.); 2 Department of Physiotherapy, College of Health Sciences, Gulf Medical University, Ajman P.O. Box 4184, United Arab Emirates; 3 Microbiology Lab, Thumbay Hospital, Ajman P.O. Box 4184, United Arab Emirates; dr.supriya@thumbaylabs.com; 4 Department of Health Sciences, College of Applied and Health Sciences, A ‘Sharqiyah University, Ibra P.O. Box 42, Oman; einas.osman@asu.edu.om; 5 College of Medical Laboratory Sciences, Omdurman Ahlia University, Omdurman P.O. Box 311119, Sudan; saharsedahmed@gmail.com

**Keywords:** efflux pump, *mef(A)*, *msr(D)*, *Streptococcus*, antimicrobial resistance, macrolide resistance

## Abstract

Background: Efflux-mediated macrolide resistance represents an emerging threat in *Streptococcus* infections globally. However, molecular epidemiological data from the Gulf region, particularly the United Arab Emirates (UAE), remain limited. This study addresses this knowledge gap by investigating efflux pump resistance mechanisms in clinical *Streptococcus* isolates. Methods: A cross-sectional study analyzed 100 clinical isolates (99 *Streptococcus* and 1 *Enterococcus*) from Thumbay Hospital, Ajman, UAE (October–December 2024). Antimicrobial susceptibility testing for minimum inhibitory concentration (MIC) determination was performed using the DxM 1096 MicroScan WalkAway system (Beckman Coulter Inc., Brea, CA, USA; LabProv4.42). PCR detected *mef(A/E)*, *msr(D)*, and *tet(K)* resistance genes with sequencing confirmation. Comparative genomic analysis was performed using a total of 30 publicly available *Streptococcus* genomes: 15 from India and 15 from Saudi Arabia. Statistical analysis employed chi-square tests, Fisher’s exact tests, and multivariate logistic regression with Bonferroni correction (α = 0.05). Results: Among the isolates, erythromycin resistance occurred in 39 isolates (39%, 95% CI: 29.4–49.2%) and clindamycin resistance in 31 isolates (31%, 95% CI: 22.1–40.9%). The *mef(A/E)* gene was detected in 31 isolates (31%, 95% CI: 22.1–40.9%), and *msr(D)* in 3 isolates (3%, 95% CI: 0.6–8.5%), with co-occurrence in 3 isolates (3%). No isolates harbored *tet(K)*. Multivariate analysis identified *mef(A/E)* as the strongest predictor of macrolide resistance (OR = 18.7, 95% CI: 7.9–44.2, *p* < 0.001). Regional comparison revealed significant differences: *mef(A/E)* prevalence was 31% (UAE), 87% (India), and 0% (Saudi Arabia) (*p* < 0.001). Conclusions: This study provides the first molecular characterization of efflux-mediated macrolide resistance in UAE *Streptococcus* isolates. The predominance of *mef(A/E)*-mediated resistance with confirmed efflux activity highlights the clinical significance of active surveillance and targeted antimicrobial stewardship in the region.

## 1. Introduction

Antimicrobial resistance (AMR) poses a critical global health challenge, with *Streptococcus* species contributing significantly through diverse resistance mechanisms affecting multiple antibiotic classes [1,2]. These Gram-positive pathogens cause infections ranging from mild pharyngitis to life-threatening invasive diseases, including sepsis, meningitis, and streptococcal toxic shock syndrome [3].

Increasing resistance to antibiotics among *Streptococcus* species, particularly resistance to macrolides, lincosamides, and tetracyclines, has been documented globally across diverse geographic regions and healthcare settings [4,5]. Macrolide resistance in *Streptococcus* occurs through two primary mechanisms: ribosomal methylation via *erm* genes (constitutive or inducible MLS_β_ phenotype) and active drug efflux via *mef* and *msr* genes (M phenotype) [6].

Efflux pumps actively transport antibiotics out of bacterial cells, lowering intracellular antibiotic concentrations to levels below therapeutic effectiveness. The *mef(A)* and *mef(E)* genes encode Major Facilitator Superfamily (MFS) transporters that confer resistance specifically to 14- and 15-membered macrolides while maintaining susceptibility to 16-membered macrolides and lincosamides, defining the M phenotype [7]. These efflux pumps function as proton-antiporter systems, utilizing the proton motive force to expel macrolide molecules from the bacterial cytoplasm before they reach their ribosomal targets [8].

The *msr(D)* gene encodes an ATP-binding cassette (ABC) transporter that functions synergistically with Mef pumps when co-expressed. This co-expression significantly enhances resistance levels to macrolides and extends the resistance spectrum to include streptogramin B antibiotics [9,10]. Recent structural and functional studies have revealed that *msr(D)* operates through ATP-dependent conformational changes, actively extruding macrolides and demonstrating substrate overlap with Mef transporters, thereby providing additive resistance when both systems are present [11,12].

The genetic organization of these resistance determinants is clinically significant. The *mef(A)* and *msr(D)* genes are frequently co-located on mobile genetic elements, including the mega element and Tn1207.1-like transposons, facilitating horizontal gene transfer between streptococcal species and geographic dissemination [13,14]. This mobile nature contributes to the rapid spread of efflux-mediated resistance in clinical settings.

The *tet(K)* gene represents another efflux mechanism, encoding an MFS transporter specific for tetracyclines. Unlike macrolide efflux systems, *tet(K)* confers resistance by actively removing tetracycline molecules from the bacterial cell, preventing their binding to the 30S ribosomal subunit [15,16]. While predominantly found in staphylococci, *tet(K)* has been occasionally reported in streptococcal isolates, warranting surveillance [17].

Understanding the molecular mechanisms and epidemiology of efflux-mediated resistance is essential for developing targeted antimicrobial stewardship strategies and preserving therapeutic options for streptococcal infections [18,19].

The incidence of macrolide resistance among *Streptococcus* species varies substantially across Middle Eastern countries. Recent surveillance studies have documented macrolide resistance rates of 15–42% for *S. pneumoniae* isolates in the region, with country-specific rates of 18% in Lebanon [20], 35% in Saudi Arabia [21], and 28% in Jordan [22]. For *S. pyogenes* (Group A *Streptococcus*), resistance rates range from 22% to 58% depending on geographic location and study period [23,24]. However, comprehensive molecular characterization of resistance mechanisms—distinguishing efflux-mediated (*mef/msr* genes) from target-site modification (*erm* genes)—remains limited for Gulf Cooperation Council countries, particularly the United Arab Emirates. This knowledge gap is particularly concerning in a region with a heterogeneous expatriate population that can introduce and spread resistance genes from diverse geographic origins.

The objectives of this study are to: (1) Determine the prevalence of efflux-mediated resistance genes *mef(A/E)*, *msr(D)*, and *tet(K)* in clinical *Streptococcus* isolates from the UAE; (2) Assess phenotypic resistance profiles and validate efflux pump activity; (3) Identify independent predictors of macrolide resistance; and (4) Compare regional resistance patterns to inform antimicrobial stewardship strategies.

## 2. Results

### 2.1. Demographic Characteristics and Antimicrobial Resistance Profile

Among 100 isolates (99 *Streptococcus* and 1 *Enterococcus*), 59 were from female patients (59%) and 41 from male patients (41%). The age distribution was as follows: 0–18 years (26 isolates, 26%), 19–35 years (49 isolates, 49%), 36–50 years (22 isolates, 22%), and 51–60 years (3 isolates, 3%). Patient demographics reflected the UAE’s diverse population: Indian (34 patients, 34%), Pakistani (21 patients, 21%), Egyptian (8 patients, 8%), UAE nationals (7 patients, 7%), and other nationalities (30 patients, 30%). Specimen distribution included throat swabs (47 isolates, 47%), vaginal swabs (30 isolates, 30%), pus samples (10 isolates, 10%), ear swabs (5 isolates, 5%), sputum (2 isolates, 2%), blood cultures (2 isolates, 2%), urethral samples (2 isolates, 2%), cerebrospinal fluid (1 isolate, 1%), and nasopharyngeal swabs (1 isolate, 1%). The distribution of bacterial species across specimen types (Table 1) demonstrates marked anatomical site specificity. Group A streptococci were overwhelmingly isolated from throat specimens (93.3% of Group A isolates), consistent with their role as a leading cause of bacterial pharyngitis. Group B streptococci predominated in vaginal specimens (83.3% of Group B isolates), reflecting colonization of the female genital tract. *S. pneumoniae* was found exclusively in respiratory specimens and normally sterile sites (blood, CSF), consistent with its pathogenic profile. Chi-square test of independence confirmed that bacterial species distribution was strongly non-random with respect to specimen type (χ^2^ = 127.4, *p* < 0.001, Cramér’s V = 0.798).

**Figure 1:**
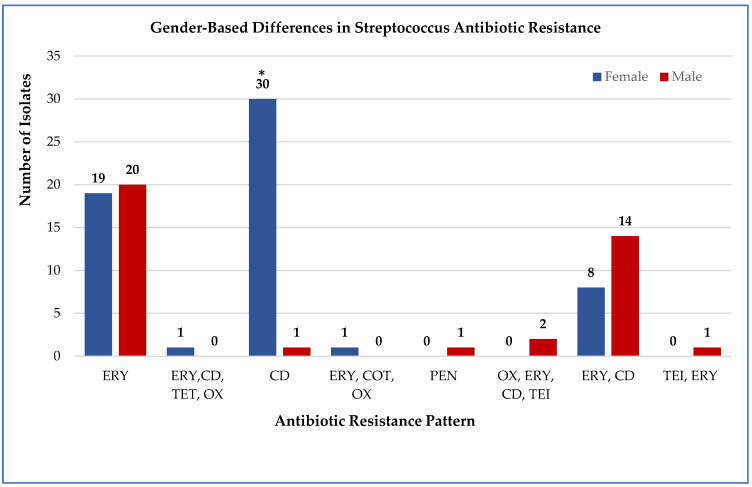
Gender-based comparison of *Streptococcus* resistance to multiple antibiotics. The proportion of resistant isolates among male (*n* = 41) and female (*n* = 59) patients. Abbreviations: ERY, erythromycin; CD, clindamycin; PEN, penicillin; OX, oxacillin; LEV, levofloxacin; TET, tetracycline; CIP, ciprofloxacin; COT, cotrimoxazole; RIF, rifampin; TEI, teicoplanin. Numbers above the bars indicate the number of resistant cases per gender. * Only clindamycin resistance differed significantly between genders (χ^2^ = 4.327, *p* = 0.038).

Across both genders, erythromycin resistance was the most frequent resistance phenotype (39 isolates, 39%), followed by clindamycin resistance (31 isolates, 31%) (Supplementary Table S3). Gender-based statistical comparisons revealed that only clindamycin resistance differed significantly between males and females (χ^2^ = 4.327, *p* = 0.038), with female patients showing higher resistance rates (21/59 vs. 10/41). Other antibiotic comparisons showed no significant gender differences: erythromycin (χ^2^ = 0.029, *p* = 0.865), penicillin (Fisher’s exact, *p* = 1.000), oxacillin (Fisher’s exact, *p* = 1.000), and levofloxacin (Fisher’s exact, *p* = 1.000). All other comparisons were not significant (*p* > 0.05) (Figure 1).

**Figure 2:**
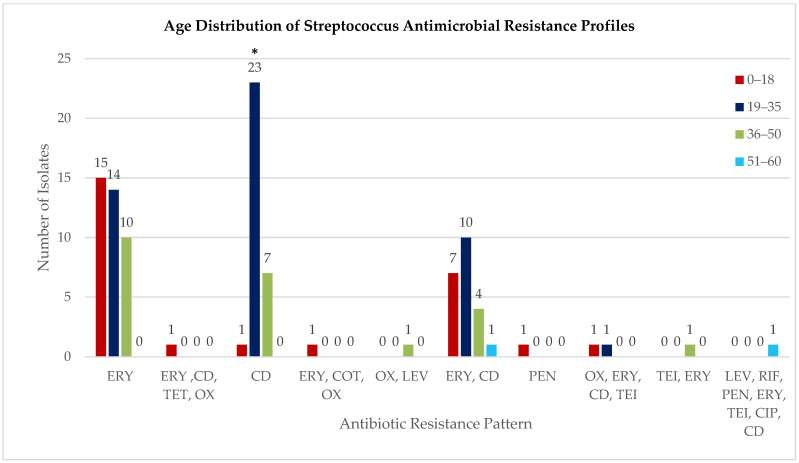
Age distribution of *Streptococcus* antimicrobial resistance profiles. Age-stratified distribution of antimicrobial resistance profiles across four age groups: 0–18 years, 19–35 years, 36–50 years, and 51–60 years. Resistance profiles on the x-axis include single-drug resistance (ERY: erythromycin; CD: clindamycin; PEN: penicillin; OX: oxacillin; LEV: levofloxacin; TET: tetracycline; CIP: ciprofloxacin; COT: cotrimoxazole; RIF: rifampin; TEI: teicoplanin) and multidrug combinations. Numbers on top of the bars indicate the count of resistant isolates per age group. Overall comparison across age groups was statistically significant (χ^2^ = 18.445, *p* = 0.018 *), with the 19–35-year group exhibiting the highest cumulative resistance burden. Data represents the distribution of resistance patterns among 100 isolates (99 *Streptococcus*, 1 *Enterococcus*).

Age-stratified analysis across all tested antimicrobials demonstrated that the 19–35 years age group exhibited the highest cumulative resistance burden, with 63.3% of isolates showing resistance to one or more antibiotics (31/49 isolates), compared to 46.2% in 0–18 years (12/26), 54.5% in 36–50 years (12/22), and 33.3% in 51–60 years (1/3). Overall distribution of resistance across age groups was statistically significant (χ^2^ = 18.445, *p* = 0.018). Age-stratified distribution of antimicrobial resistance profiles across four age groups: 0–18 years, 19–35 years, 36–50 years, and 51–60 years. Resistance profiles on the x-axis include single-drug resistance (ERY: erythromycin; CD: clindamycin; PEN: penicillin; OX: oxacillin; LEV: levofloxacin; TET: tetracycline; CIP: ciprofloxacin; COT: cotrimoxazole; RIF: rifampin; TEI: teicoplanin) and multidrug combinations. Numbers on top of the bars indicate the count of resistant isolates per age group. Overall comparison across age groups was statistically significant (χ^2^ = 18.445, *p* = 0.018, *p* < 0.05 *), with the 19–35-year group exhibiting the highest cumulative resistance burden (Figure 2).

### 2.2. Species-Specific Antimicrobial Resistance Profiles

Isolates were classified into Lancefield groups (A, B, C, F, G) using latex agglutination with the Prolex™ Streptococcal Grouping Latex Kit (Pro-Lab Diagnostics Inc., Rochester/Bromborough, NY/Merseyside, USA/UK; IFU PL030_en05), and *S. pneumoniae* was confirmed by optochin sensitivity (≥14 mm inhibition zone), as described in Section 2.1.

The single *Enterococcus* isolate demonstrated universal resistance (100%) to all tested antibiotics including clindamycin, ciprofloxacin, erythromycin, levofloxacin, penicillin, rifampin, and tetracycline. Among the 99 *Streptococcus* isolates: Group A streptococci exhibited complete resistance to erythromycin (100%) and partial resistance to clindamycin (37.8%). Group B isolates showed high resistance to clindamycin (91.6%) and low erythromycin resistance (16.7%). Group C displayed complete resistance to clindamycin (100%). Group F isolates were fully resistant to erythromycin (100%). Group G displayed complete resistance to erythromycin (100%) but lower resistance to clindamycin (20%). *S. pneumoniae* isolates exhibited variable resistance, with highest rates for oxacillin (66.7%) and erythromycin (66.7%), while resistance to ciprofloxacin and tetracycline was lower (16.7% each). These findings highlight both species-specific and antibiotic-specific patterns (Table 2).

### 2.3. Antimicrobial Resistance Profiles

Resistance rate was highest for erythromycin (39 isolates, 39%; 95% CI: 29.4–49.2%) and clindamycin (31 isolates, 31%; 95% CI: 22.1–40.9%). Combined erythromycin-clindamycin resistance occurred in 22 isolates (22%; 95% CI: 14.3–31.4%). Multidrug resistance (≥3 antibiotic classes) was observed in 8 isolates (8%; 95% CI: 3.5–15.2%). The single *Enterococcus* isolate displayed multidrug resistance. Among Group A streptococci (*n* = 45), resistance was split between erythromycin alone (62.2%) and combined erythromycin-clindamycin resistance (37.8%). Group B streptococci (*n* = 36) showed predominantly clindamycin resistance (83.3%), with smaller proportions exhibiting combined resistance (8.33%) or remaining Multidrug resistance (2.78%). In Group G streptococci (*n* = 10), erythromycin resistance alone was most frequent (80%). The six *S. pneumoniae* isolates included four (66.7%) that were multidrug resistant (Table 3).

### 2.4. Efflux Pump Activity Validation Using Ethidium Bromide Accumulation

Beyond genetic detection of efflux pump genes, we performed functional validation using ethidium bromide (EtBr) accumulation assays. Ethidium bromide serves as a fluorescent substrate for efflux pumps; cells with active efflux mechanisms accumulate less dye and exhibit reduced fluorescence compared to cells lacking efflux activity.

Antibiotic-sensitive *Streptococcus* isolates demonstrated concentration-dependent EtBr accumulation. Fluorescence intensity increased progressively from 14,500 ± 1200 relative fluorescence units (RFU) at 0.5 μg/mL EtBr to 32,150 ± 2840 RFU at 4.0 μg/mL EtBr, indicating passive diffusion-driven dye accumulation without significant active efflux.

**Figure 3:**
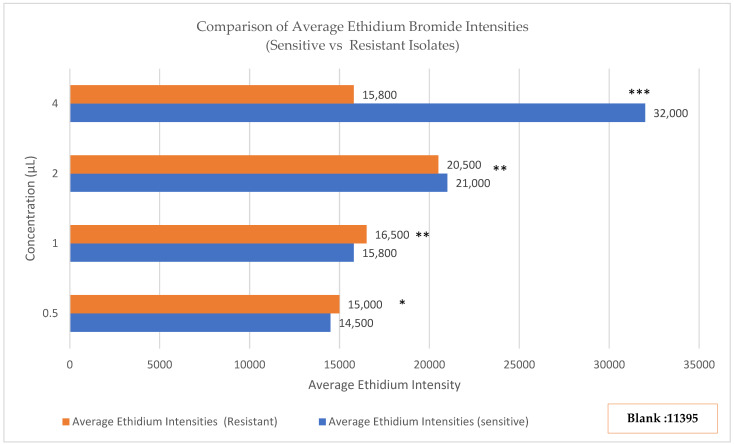
Ethidium bromide (EtBr) accumulation assay in sensitive and resistant *Streptococcus* isolates. Fluorescence increased with EtBr concentration in sensitive isolates (0.5 μg/mL: ~14,500 RFU; 4.0 μg/mL: 32,150 ± 2840 RFU) but plateaued and declined in resistant isolates (0.5 μg/mL: ~15,000 RFU; 4.0 μg/mL: 16,420 ± 2650 RFU). Background fluorescence (EtBr + PBS) was 11,395 RFU. Statistical significance was assessed by Mann–Whitney U test: 0.5 μg/mL (*p* = 0.024, *), 1.0 μg/mL (*p* = 0.003, **), 2.0 μg/mL and 4.0 μg/mL (*p* < 0.001, ***), indicating progressively greater differences at higher EtBr concentrations, consistent with inducible efflux pump activity.

In contrast, antibiotic-resistant isolates exhibited markedly different kinetics. Fluorescence rose initially from 15,000 ± 1400 RFU at 0.5 μg/mL to a plateau of 20,680 ± 3120 RFU at 2.0 μg/mL, then paradoxically declined to 16,420 ± 2650 RFU at 4.0 μg/mL. This concentration-dependent fluorescence reduction at higher substrate loads is characteristic of saturable efflux pump activity, where increased substrate availability triggers enhanced pump function (Figure 3). Statistical comparison using Mann–Whitney U tests confirmed significant differences between sensitive and resistant isolates at all EtBr concentrations: 0.5 μg/mL (*p* = 0.024), 1.0 μg/mL (*p* = 0.003), 2.0 μg/mL (*p* < 0.001), and 4.0 μg/mL (*p* < 0.001). The magnitude of difference increased with substrate concentration, consistent with inducible efflux pump expression. These functional assays provide direct biochemical evidence that *mef(A/E)* genes encode active, functional efflux pumps in our clinical isolates rather than silent genetic elements.

### 2.5. Detection of Efflux Resistance Genes (PCR)

**Figure 4:**
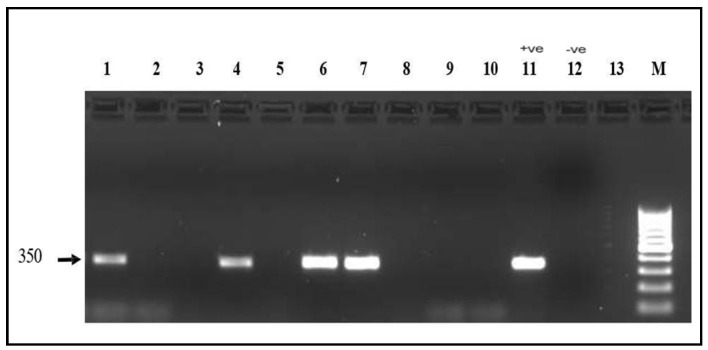
*mef(A)* gene electrophoresis result Detection of the *mef(A)* gene (350 bp) on a 1.5% agarose gel. Lanes 1, 4, 6, and 7: positive samples. Lanes 2, 3, 5, 8, 9, 10 and 13: Negative samples; Lane 11: positive control; Lane 12: Negative control; M: 100 bp molecular marker.

**Figure 5:**
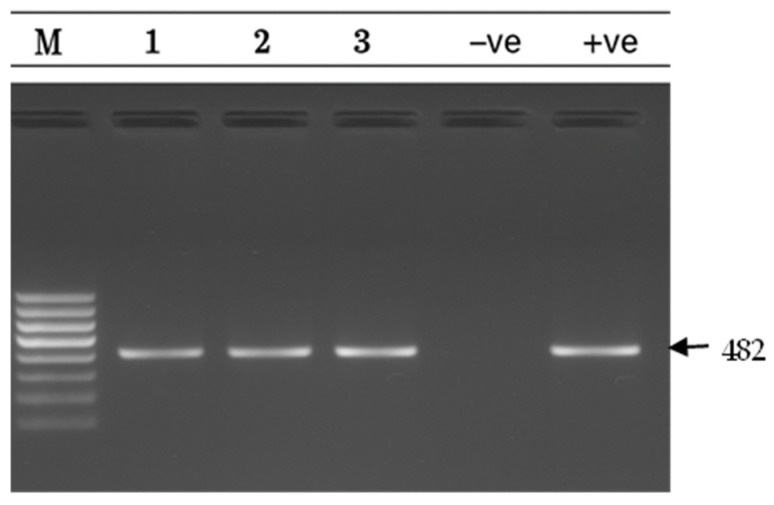
*msr(D)* gel electrophoresis result *msr(D)* gene detection (482 bp) on a 1.5% agarose gel. Lane M: 100 bp DNA ladder; Lanes 1–3: positive samples; Lane −ve: Negative control; Lane +ve: positive control.

PCR analysis revealed *mef(A/E)* in 31 isolates (31%; 95% CI: 22.1–40.9%) (Figure 4) and *msr(D)* in 3 isolates (3%; 95% CI: 0.6–8.5%) (Figure 5). No isolates harbored *tet(K)*. Co-occurrence of *mef(A/E)* and *msr(D)* was observed in all 3 *msr(D)*-positive isolates (3%).

### 2.6. Genotype–Phenotype Correlation

Analysis using the φ (phi) coefficient showed a strong positive correlation (φ = 0.672, *p* < 0.001), indicating that isolates carrying *mef(A/E)* were much more likely to be resistant to erythromycin. Of the 31 *mef(A/E)*-positive isolates, 29 (93.5%) were resistant to erythromycin, while among the 69 isolates without the gene, only 10 (14.5%) were resistant. This marked difference highlights the importance of *mef(A/E)* in conferring macrolide resistance. Isolates with the gene were almost always resistant, whereas most isolates lacking it remained susceptible, supporting a role for efflux-mediated mechanisms in erythromycin resistance.

### 2.7. Predictors of Macrolide Resistance in Clinical Isolates

The *mef(A/E)* gene was the strongest predictor, increasing the likelihood of resistance nearly 19-fold (adjusted OR 18.7, 95% CI: 7.9–44.2, *p* < 0.001). Group A *Streptococcus* infections were also strongly associated with resistance (adjusted OR 12.4, 95% CI: 5.2–29.6, *p* < 0.001). Other factors associated with higher risk included female patients (adjusted OR 2.9, 95% CI: 1.3–6.8, *p* = 0.012), individuals aged 19–35 years (adjusted OR 2.3, 95% CI: 1.1–4.9, *p* = 0.028), and throat specimens (adjusted OR 3.1, 95% CI: 1.4–6.9, *p* = 0.005). These results show that both bacterial characteristics and host factors are independently associated with macrolide resistance (Table 4). The model demonstrated good performance, explaining 74.2% of the variability in macrolide resistance (Nagelkerke R^2^ = 0.742) and showing adequate fit to the data (Hosmer–Lemeshow test, *p* = 0.456).

### 2.8. Regional Comparison of Antibiotic Resistance Patterns

A comparative analysis of antibiotic resistance patterns and resistance gene profiles among *Streptococcus* species isolates from the UAE, Saudi Arabia, and India revealed significant regional variations in both genotypic and phenotypic characteristics.

**Figure 6:**
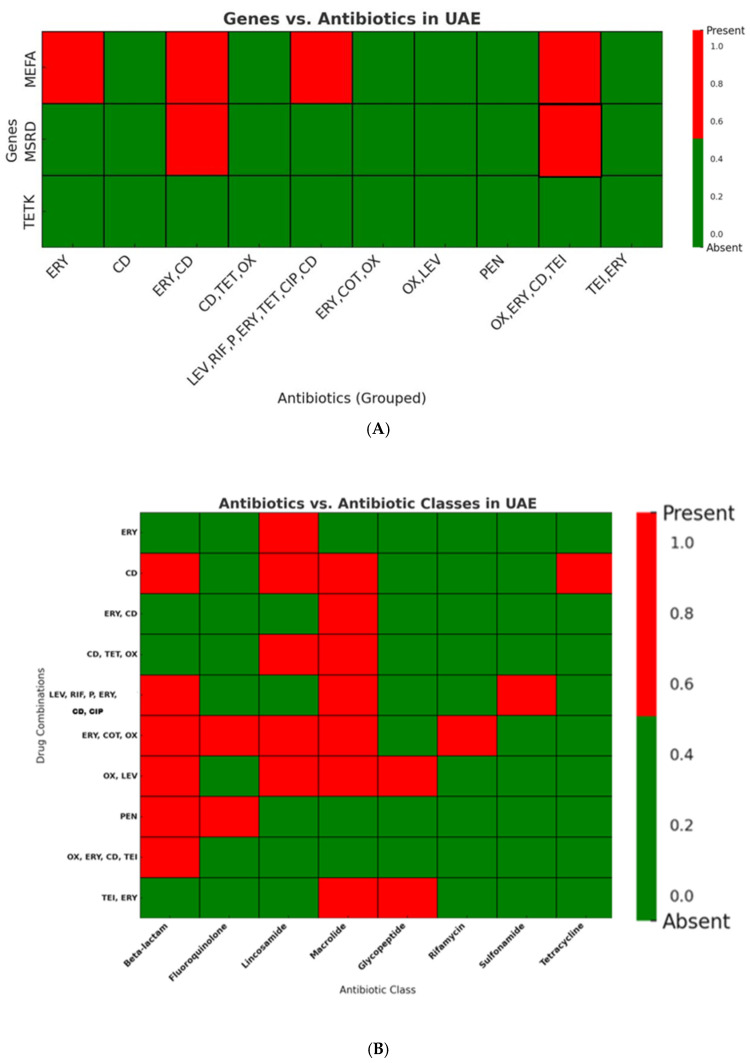
(**A**): Distribution of Resistance Genes in UAE *Streptococcus* Isolates. The macrolide resistance gene *mef(A)* was frequently detected, while *msr(D)* was observed in only a few isolates. The tetracycline resistance gene *tet(K)* was absent in all UAE isolates (ERY: erythromycin; CD: clindamycin; PEN: penicillin; OX: oxacillin; LEV: levofloxacin; TET: tetracycline; CIP: ciprofloxacin; COT: cotrimoxazole; RIF: rifampin; TEI: teicoplanin). Heatmaps illustrate the presence (Red) and absence (Green) of antibiotic classes across tested drug. (**B**): Distribution of Antibiotic Resistance Classes in UAE *Streptococcus* Isolates. Macrolide resistance was the most prevalent, followed by resistance to β-lactams, while tetracycline resistance was detected only in a single group. Resistance was distributed across various antibiotic combinations, mainly involving macrolides, lincosamides, and β-lactams (ERY: erythromycin; CD: clindamycin; PEN: penicillin; OX: oxacillin; LEV: levofloxacin; TET: tetracycline; CIP: ciprofloxacin; COT: cotrimoxazole; RIF: rifampin; TEI: teicoplanin).

In UAE isolates (*n* = 100), resistance was most pronounced against macrolides (68%) and lincosamides (56%), while resistance to other antibiotic classes, including tetracyclines, beta-lactams, and fluoroquinolones, was minimal. This phenotypic profile corresponded closely with genomic data, where *mef(A)* was detected in 31% of isolates and *msr(D)* in 3%, whereas *tet(K)* was absent (Figure 6A,B; Supplementary Figure S1).

**Figure 7:**
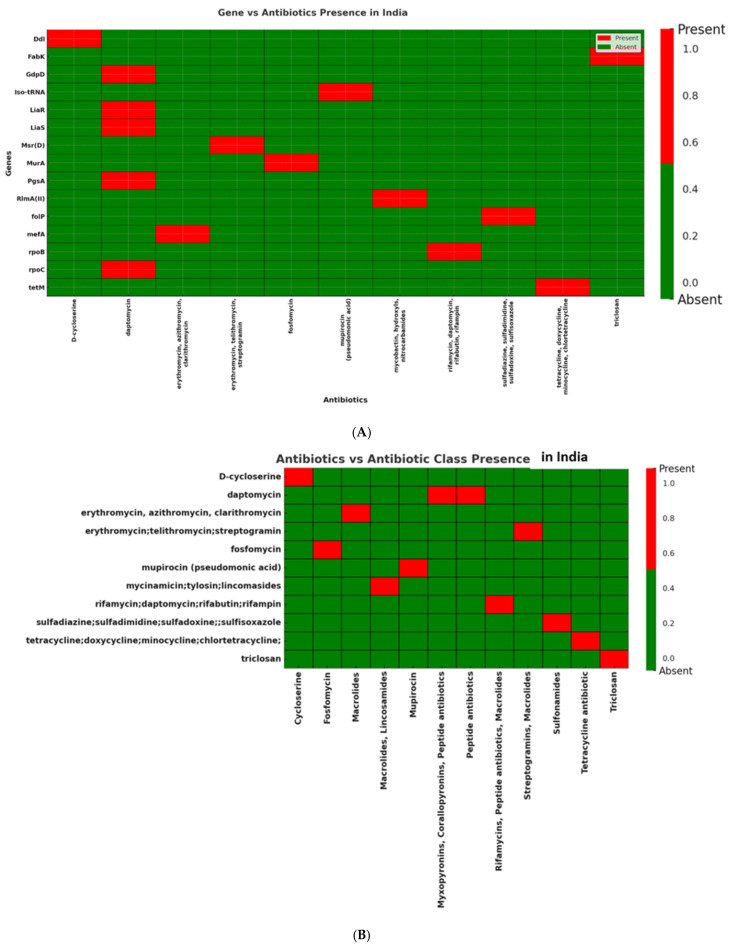
(**A**): Distribution of antibiotic resistance genes in *Streptococcus* isolates from India. Presence of *mef(A)* and *msr(D)* genes, confirming macrolide resistance against erythromycin, azithromycin, and clarithromycin. In addition, the Indian dataset displayed a broader resistance profile with *tet(M)*, *rpoC*, and *liaS*, associated with resistance to tetracycline, rifampin, and daptomycin, respectively, indicating a diverse distribution of resistance determinants in Indian clinical isolates. (**B**): Distribution of antibiotic classes in *Streptococcus* isolates from India. Several antibiotic classes including macrolides, peptide antibiotics, rifamycins, and tetracyclines are represented among the Indian isolates, reflecting a broad spectrum of resistance determinants.

Indian isolates (*n* = 15) exhibited more variable resistance patterns, affecting multiple antibiotic classes including macrolides, tetracyclines, glycopeptides, sulfonamides, peptide antibiotics, and rifamycins. *Mef(A)* (87%) and *msr(D)* (87%), as well as other resistance genes including *tet(M)*, *rpoC*, and *liaS*, were highly prevalent in these isolates (Figure 7A,B; Supplementary Figure S1).

**Figure 8:**
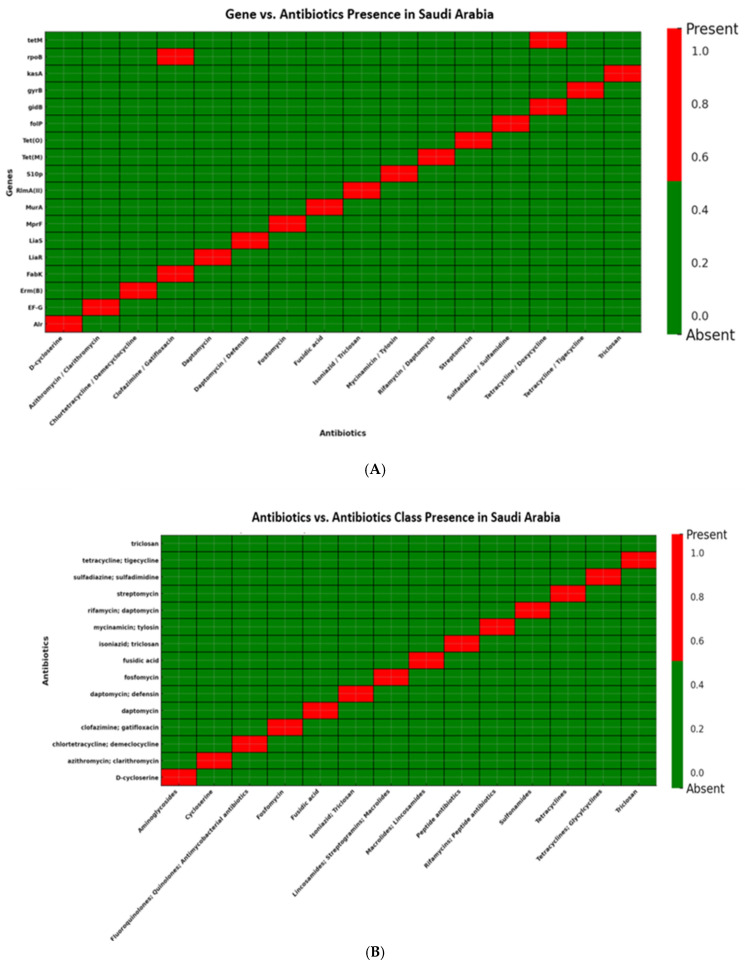
(**A**): Distribution of antibiotic resistance genes in *Streptococcus* isolates from Saudi Arabia. Presence of *erm(B)* and *tet(M)*, conferring resistance to lincosamides and tetracyclines, respectively. Additional genes detected included *gyrB* and *folP* (fluoroquinolone and sulfonamide resistance) as well as *liaS* and *mprF* (cell envelope and peptide antibiotic resistance). Notably, none of the isolates carried *mef(A)*, *msr(D)*, or *tet(K)*, indicating the absence of efflux-mediated resistance mechanisms in the Saudi Arabian dataset. (**B**): Distribution of antibiotic classes in *Streptococcus* isolates from Saudi Arabia. Resistance was observed in lincosamides and tetracyclines, supported by the presence of *erm(B)* and *tet(M)*. Additional resistance was noted in fluoroquinolones and sulfonamides through *gyrB* and *folP*, as well as peptide antibiotics via *liaS* and *mprF*. Notably, none of the isolates showed efflux-mediated resistance, as *mef(A)*, *msr(D)*, and *tet(K)* were absent.

Saudi Arabian isolates (*n* = 15) displayed resistance in fewer antibiotic classes and fewer drugs per class. These isolates were positive for *erm(B)*, *tet(M)*, *liaS*, *folP*, and *mprF*, suggesting that target-site mutations, rather than efflux mechanisms, are predominant contributors to resistance in this region (Figure 8A,B; Supplementary Figure S1).

Statistical analysis confirmed that differences in prevalence of *mef(A)* (*p* < 0.001) and *msr(D)* (*p* < 0.001) were significant across countries. *tet(K)* remained absent in all regions (*p* = 1.0). Fisher’s exact test with Bonferroni correction (α = 0.0125) confirmed that *mef(A/E)*, *msr(D)*, and *erm(B)* prevalence differed significantly between countries (*p* < 0.0125) (Supplementary Table S4).

Resistance also varied across antibiotic classes. Macrolide resistance prevalence: 68% UAE (68/100), 86.7% India (13/15), and 80% Saudi Arabia (12/15). While UAE showed a lower proportion, the absolute number was higher due to a larger sample size. Lincosamide resistance: UAE 56%, India 6.7%, and Saudi Arabia 20%. Tetracycline resistance: UAE 1%, India 40%, and Saudi Arabia 33.3% (Supplementary Figure S2). Pairwise Fisher’s exact tests showed significant differences for tetracyclines (UAE vs. India, *p* < 0.001; UAE vs. Saudi, *p* < 0.001) and lincosamides (UAE vs. India, *p* < 0.001; UAE vs. Saudi, *p* = 0.012), while macrolide differences were not significant (all *p* > 0.05) (Supplementary Table S5).

## 3. Materials and Methods

A cross-sectional study was conducted at Thumbay Hospital, Ajman, UAE, between October and December 2024. The study protocol received approval from the Gulf Medical University Institutional Review Board (IRB-COHS-STD-76-October-2024). A total of 100 clinical isolates were collected from different samples, including respiratory specimens, wound swabs, blood cultures, urogenital samples, and sterile body fluids. Inclusion criteria required: (1) confirmed *Streptococcus* identification, (2) clinical significance, and (3) viable isolate for molecular analysis.

### 3.1. Bacterial Identification and Characterization

During the study period, 100 consecutive isolates of Gram-positive cocci in chains were collected based on preliminary Gram-stain morphology. Organisms were isolated on 5% sheep blood agar and chocolate agar (5% CO_2_, 37 °C). Final identification revealed 99 *Streptococcus* isolates (Lancefield groups A, B, C, F, G, and *S. pneumoniae*) and 1 *Enterococcus* isolate. Given that both genera are catalase-negative Gram-positive cocci with similar preliminary characteristics and clinical relevance, the *Enterococcus* isolate was retained to reflect real-world clinical microbiology practice and provide comprehensive context for resistance patterns at the study institution. All 100 isolates underwent antimicrobial susceptibility testing. For molecular analyses of *mef(A/E)*, *msr(D)*, and *tet(K)* genes—primarily characterized in *Streptococcus* species—the *Enterococcus* isolate was tested but interpreted separately given the different efflux pump mechanisms typical of this genus.

Streptococcal grouping for Lancefield groups A, B, C, F, and G was performed using the Prolex™ Streptococcal Grouping Latex Kit (Pro-Lab Diagnostics Inc., Rochester/Bromborough, NY/Merseyside, USA/UK; IFU PL030_en05). *S. pneumoniae* identification was confirmed by optochin sensitivity (≥14 mm inhibition zone). Identification was performed at the Lancefield group level for several reasons: (1) standard clinical laboratory workflow provides sufficient information for therapeutic decisions; (2) rapid and cost-effective approach is suitable for surveillance studies with 100 isolates; (3) Lancefield grouping correlates well with species for beta-hemolytic streptococci (Group A = *S. pyogenes*, Group B = *S. agalactiae*); (4) Lancefield group identification is sufficient according to CLSI in order to perform the AST. *S. pneumoniae* was identified to species level using optochin sensitivity as standard practice. This pragmatic approach reflects real-world clinical laboratory practice while maintaining clinically relevant categorization. All procedures followed CLSI guidelines M100, 33rd edition-2023 [25].

### 3.2. Antimicrobial Susceptibility Testing

Antimicrobial susceptibility was determined using the DxM 1096 MicroScan Walk way system (Beckman Coulter Inc., Brea, CA, USA; LabPro v4.42) with panels (Beckman Coulter Inc., Brea, CA, USA, LabPro v4.42). The panel tested 28 antimicrobials across multiple classes: Beta-lactams (penicillin, ampicillin, ceftriaxone, cefotaxime); Macrolides (erythromycin, azithromycin, clarithromycin); Lincosamides (clindamycin); Fluoroquinolones (levofloxacin, moxifloxacin, ciprofloxacin); Tetracyclines (tetracycline, doxycycline); Glycopeptides (vancomycin, teicoplanin); Aminoglycosides (gentamicin); Folate pathway inhibitors (trimethoprim–sulfamethoxazole); Oxazolidinones (linezolid); Lipopeptides (daptomycin); and Others (chloramphenicol, rifampin, nitrofurantoin, quinupristin–dalfopristin). Minimum inhibitory concentrations (MICs) were interpreted according to CLSI guidelines [25]. Quality control followed IQCP standards. Quality control strains included *S. pneumoniae* ATCC 49619, *S. pyogenes* ATCC 19615, *S. agalactiae* ATCC 13813, and *S. mitis* ATCC 49456.

### 3.3. Assessment of Efflux Pump Activity via Ethidium Bromide Accumulation Assay

Isolates of *Streptococcus* species, including both antibiotic-resistant and sensitive strains, were grown in Brain Heart Infusion (BHI) broth at 37 °C for 4–6 h to reach active growth. Cultures were adjusted to an OD_600_ of 0.2–0.3 using a Color Wave CO7500 Colorimeter (Biochrom Ltd., Cambridge, UK; Spreadsheet Interface Software, Part No. 80211223), indicating mid-log phase. To assess efflux activity, ethidium bromide (EtBr) was prepared in sterile PBS at concentrations of 0.5, 1.0, 2.0, and 4.0 μg/mL. Cultures were centrifuged at 5000× *g* for 5 min, the supernatant discarded, and the pellets resuspended in 100 µL of the respective EtBr dilution. These suspensions were transferred into 96-Well Microtiter Plates (Corning Inc., Corning, NY, USA) and incubated at 37 °C. Fluorescence was measured using a Bio-Rad xMark Microplate Reader (Bio-Rad Laboratories Inc., Hercules, CA, USA; Microplate Manager™, check lab version) (excitation 530 nm, emission 600 nm) at 15, 25, 30, and 45 min intervals. Background fluorescence was corrected using a blank well containing only EtBr and PBS. All measurements were performed in biological triplicates, with each isolate tested three times to ensure reproducibility, alongside appropriate controls.

### 3.4. Molecular Detection of Resistance Genes

#### 3.4.1. DNA Extraction, PCR Amplification, and Agarose Gel Electrophoresis

Genomic DNA was extracted using the GeneJET Genomic DNA Purification Kit (Thermo Fisher Scientific Inc., Waltham, MA, USA; Kit Revision 6, manual) PCR targeted *mef(A/E)*, *msr(D)*, and *tet(K)* genes using validated primers [26,27] (Table 5). Each reaction was carried out in a final volume of 25 µL, consisting of template DNA and a master mix containing forward and reverse primers (10 µM) synthesized by e-Oligos, Taq DNA polymerase, dNTP mix (10 mM), 10X PCR buffer, and MgCl_2_, with nuclease-free water added to reach the final volume. The thermal cycling conditions consisted of an initial denaturation at 95 °C for 5 min, followed by 35 cycles of denaturation at 95 °C for 30 s, annealing at 55 °C for 30 s, and extension at 72 °C for 45 s. A final extension step was performed at 72 °C for 5 min. PCR products were analyzed on a 1.5% agarose gel prepared in TAE buffer. Positive and negative controls were included to validate amplification results.

#### 3.4.2. Comparative Genomic Analysis of *Streptococcus* Isolates

A total of 30 clinical *Streptococcus* isolates were included in comparative genomic analysis. Initially, genomic data from the UAE were planned for comparison; however, no publicly available *Streptococcus* datasets from the UAE were identified. Publicly available genome sequences from India (*n* = 15) and Saudi Arabia (*n* = 15) were retrieved from NCBI (see Supplementary Tables S1 and S2 for reference strains). Genome annotation was performed using PATRIC v3.34.11, incorporating the RAST toolkit for gene identification and characterization. Antimicrobial resistance (AMR) genes were identified using ResFinder 4.0 and the CARD database, with thresholds set at ≥90% identity and ≥60% coverage. In addition, a k-mer-based method was employed to predict resistance mechanisms, determine affected antibiotic classes, and identify specific resistance genes present. All genomic analyses were conducted using sequences from National Center for Biotechnology Information (NCBI). (https://www.ncbi.nlm.nih.gov/)—Accessed on 1 March 2025. (Supplementary Tables S1 and S2).

### 3.5. Statistical Analysis

Data analysis was performed using IBM SPSS v29.0, with descriptive statistics including frequencies, percentages, and 95% confidence intervals. Chi-square and Fisher’s exact tests were applied to evaluate associations between categorical variables, with significance level set at *p* < 0.05. For antibiotic classes, chi-square tests with pairwise Fisher’s exact tests were used. For resistance genes, only overall tests were applied due to highly skewed data with many zero values. Multivariate logistic regression identified independent predictors while controlling for age, gender, specimen type, and bacterial species. Bonferroni correction was applied for multiple comparisons, and effect sizes were calculated using Cramér’s V. Comparisons between antibiotic-sensitive and resistant isolates in efflux assays were performed using the Mann–Whitney U test, as fluorescence data were not normally distributed. A *p*-value < 0.05 was considered statistically significant. Heat maps were created using a coding scheme (1 = presence, 0 = absence) with percentages. Data visualization was performed using Microsoft Excel Tableau Desktop (Tableau Software, Seattle, WA, USA; Tableau Desktop v2025.2.4) and Julius AI (Julius AI, San Francisco, CA, USA; Julius AI v1.0.45, released May 2025).

## 4. Discussion

This study provides the first comprehensive molecular characterization of efflux-mediated macrolide resistance in clinical *Streptococcus* isolates from the UAE. Key findings include: (1) moderate prevalence of *mef(A/E)*-mediated resistance (31%), (2) low *msr(D)* co-resistance (3%), (3) absence of *tet(K)*-mediated tetracycline efflux, (4) functional validation of efflux activity through EtBr accumulation assays, and (5) distinct regional resistance patterns compared to neighboring countries.

The predominance of *mef(A/E)*-mediated resistance conferring the M phenotype has direct therapeutic implications for UAE clinicians. Unlike *erm*-mediated MLS_β_ resistance which confers cross-resistance to macrolides, lincosamides, and streptogramin B antibiotics, the M phenotype preserves clindamycin susceptibility. For penicillin-allergic patients with Group A streptococcal pharyngitis, clindamycin remains a viable alternative despite macrolide resistance. However, clinicians should be aware that co-carriage of *mef(A/E)* and *msr(D)* genes (observed in 3% of isolates) can extend resistance to include streptogramin B antibiotics, potentially compromising combination therapies.

The high prevalence of efflux-mediated resistance (31% overall, 100% in Group A isolates) suggests that empirical macrolide therapy for suspected streptococcal infections in the UAE carries significant risk of treatment failure. Local antimicrobial guidelines should consider these resistance patterns when developing recommendations for empirical therapy.

The predominance of *mef(A/E)* over *msr(D)* in UAE isolates contrasts with patterns observed in some Asian countries, where co-occurrence is more common [28,29]. Our finding that *msr(D)* occurred exclusively with *mef(A/E)* supports previous observations that these genes are co-located on mobile genetic elements, with *msr(D)* providing enhanced resistance when co-expressed [9].

The exclusive detection of *mef(A)* and *mef(E)* variants provides valuable epidemiological insight. Both variants confer the M phenotype (resistance to 14- and 15-membered macrolides while maintaining susceptibility to 16-membered macrolides and clindamycin), unlike *erm*-mediated resistance affecting all MLS antibiotics [6]. This pattern suggests potential therapeutic alternatives for UAE patients.

The striking regional differences observed—efflux-mediated resistance predominating in UAE, target-site modification (*erm* genes) in Saudi Arabia, and mixed mechanisms in India—likely reflect complex interactions between antibiotic selection pressure, clonal spread, and horizontal gene transfer dynamics. Recent surveillance data from the Gulf region [30,31] have reported increasing macrolide consumption rates, particularly azithromycin prescribing respiratory tract infections. The UAE’s diverse expatriate population, with 34% of our study participants of Indian origin, may facilitate introduction of resistance determinants from South Asia where *mef(A/E)* prevalence is high.

The absence of *tet(K)* in our isolates aligns with previous reports showing that tetracycline resistance in streptococci is primarily mediated by ribosomal protection proteins encoded by *tet(M)* and *tet(O)* rather than efflux mechanisms [32,33]. These findings redirect attention toward *tet(M)* and *tet(O)* as more relevant surveillance targets for tetracycline resistance monitoring in *Streptococcus* populations.

The identification of female gender, young adults (19–35 years), and throat specimens as independent risk factors provides actionable insights for targeted surveillance and empirical therapy decisions. The strong association with Group A *Streptococcus* (adjusted OR = 12.4) aligns with global trends of increasing macrolide resistance in this species [34].

The elevated resistance burden in the 19–35 years age group may reflect higher healthcare utilization, acute infection rates, or occupational exposures. However, our study did not collect individual antibiotic exposure histories; this interpretation remains hypothesis-generating pending prospective investigation with detailed exposure assessment.

### 4.1. Study Limitations

Several limitations warrant consideration. First, the cross-sectional design precludes assessment of temporal trends or establishment of causal relationships between risk factors and resistance outcomes. Our multivariate analysis identifies statistical associations but cannot establish causation; prospective cohort studies with detailed exposure assessment would be required to establish causal pathways.

Second, identification at Lancefield group level rather than species level, while pragmatic and clinically relevant, may mask species-level resistance heterogeneity within groups. Third, we did not collect individual antibiotic exposure histories, limiting interpretation of age- and gender-related resistance patterns. Fourth, the comparative genomic analysis relied on publicly available sequences with potential selection bias—isolates uploaded to NCBI may not represent population-level prevalence. Fifth, our sample derives from a single tertiary care hospital in Ajman; generalizability to other UAE emirates or healthcare settings requires confirmation through multicenter surveillance.

### 4.2. Future Directions

Several research priorities emerge from this study. Whole-genome sequencing of UAE isolates would comprehensively characterize mobile genetic elements, assess clonal versus horizontal gene transfer, and identify additional resistance mechanisms beyond those targeted here. Longitudinal surveillance incorporating multiple healthcare facilities across UAE emirates would establish temporal trends and geographic variation. Prospective cohort studies with detailed antibiotic exposure assessment could establish causal relationships between antimicrobial use patterns and resistance emergence. Integration of clinical outcome data would determine whether efflux-mediated resistance impacts treatment failure rates and patient outcomes.

The robust genotype–phenotype correlation (φ = 0.672, *p* < 0.001) aligns with previous investigations establishing *mef(A/E)* as a highly predictive marker [28,29]. This consistency across diverse geographic settings validates *mef(A/E)* detection as a reliable molecular epidemiological tool and potential target for rapid diagnostic development.

The moderate prevalence of efflux-mediated resistance and emerging multidrug resistance patterns underscores the need for enhanced antimicrobial surveillance in the UAE. The regional variations observed emphasize that extrapolation of resistance data between countries may be inappropriate, supporting development of UAE-specific treatment guidelines.

## 5. Conclusions

This cross-sectional study from a single UAE tertiary care hospital detected *mef(A/E)*-mediated efflux pump genes in 31% (31/100) of clinical *Streptococcus* isolates, with functional validation demonstrating active efflux activity (*p* < 0.001). Multivariate analysis identified *mef(A/E)* as the strongest independent predictor of macrolide resistance (OR = 18.7, 95% CI: 7.9–44.2). Comparison with publicly available genomic data revealed significant regional variation in resistance mechanisms: *mef(A/E)* prevalence was 31% (UAE), 87% (India), and 0% (Saudi Arabia) (*p* < 0.001), suggesting different evolutionary trajectories. These findings provide baseline data for the UAE and indicate that multicenter surveillance incorporating diverse geographic regions and longitudinal follow-up is needed to establish population-level prevalence, temporal trends, and the clinical impact of efflux-mediated resistance on treatment outcomes.

## Figures and Tables

**Table 1:** Distribution of Bacterial Groups/Species Across Specimen Types.

Specimen Type	Group A	Group B	Group G	*S. pneumoniae*	*Enterococcus*	Group C/F	Total
Throat swab	42 (93.3%)	2 (5.7%)	3 (30%)	0	0	0	47
Vaginal swab	0	30 (83.3%)	0	0	0	0	30
Pus/wound	2 (4.4%)	2 (5.7%)	4 (40%)	1 (16.7%)	0	2 (100%)	11
Ear swab	1 (2.2%)	0	3 (30%)	1 (16.7%)	0	0	5
Respiratory *	0	0	0	2 (33.3%)	0	0	2
Blood culture	0	1 (2.8%)	0	1 (16.7%)	1 (100%)	0	3
Other **	0	1 (2.8%)	0	1 (16.7%)	0	0	2
Total	45	36	10	6	1	2	100

* Respiratory: sputum samples; ** Other: urethral, CSF, nasopharyngeal swabs. Percentages represent proportion of each bacterial group found in each specimen type. χ^2^ = 127.4, df = 42, *p* < 0.001, Cramér’s V = 0.798.

**Table 2:** Species-Specific Antimicrobial Resistance.

Species	*n*	ERY *n* (%)	CD *n* (%)	TET *n* (%)	Other Antibiotics *n* (%)
*Enterococcus*	1	1 (100)	1 (100)	--	CIP 1 (100); LEV 1 (100); PEN 1 (100); RIF 1 (100); TEI 1 (100)
Group A Strep	45	45 (100)	17 (37.8)	--	--
Group B Strep	36	6 (16.7)	33 (91.6)	--	TEI 1 (2.9)
Group C Strep	1	--	1 (100)	--	--
Group F Strep	1	1 (100)	--	--	--
Group G Strep	10	10 (100)	2 (20)	--	--
*S. pneumoniae*	6	4 (66.7)	3 (50)	1 (16.7)	OX 4 (66.7); COT 1 (16.7); TEI 2 (33.3); LEV 1 (16.7); PEN 1 (16.7)

ERY = erythromycin; CD = clindamycin; TET = tetracycline; CIP = ciprofloxacin; LEV = levofloxacin; PEN = penicillin; RIF = rifampin; TEI = teicoplanin; OX = oxacillin; COT = cotrimoxazole. *Enterococcus* (N = 1) included for descriptive purposes; represents 1% of total isolates.

**Table 3:** Comparative Resistance Patterns Across *Streptococcus* Groups.

Group/Species	*n*	Dominant Pattern	ERY Alone	CD Alone	ERY + CD	MDR (≥3)
Group A	45	ERY-dominant	28 (62.2%)	0	17 (37.8%)	0
Group B	36	CD-dominant	2 (5.56%)	30 (83.3%)	3 (8.33%)	1 (2.78%)
Group G	10	ERY-dominant	8 (80%)	0	2 (20%)	0
*S. pneumoniae*	6	MDR	0	0	3 (50%)	4 (66.7%)
Group C/F	2	Variable	1 (50%)	1 (50%)	0	0
*Enterococcus*	1	MDR	N/A	N/A	N/A	1 (100%)

ERY = erythromycin; CD = clindamycin; MDR = multidrug resistant (≥3 antimicrobial classes) Dominant Pattern summarizes the most common resistance phenotype for each group MDR was defined as resistance to ≥3 antimicrobial classes. “Other” includes isolates resistant to fewer than 3 classes. N/A = not available.

**Table 4:** Independent Predictors of Macrolide Resistance.

Variable	Adjusted OR	95% CI	*p*-Value
*mef(A/E)* positive	18.7	7.9–44.2	<0.001 ***
Group A *Streptococcus*	12.4	5.2–29.6	<0.001 ***
Female gender	2.9	1.3–6.8	0.012 *
Age 19–35 years	2.3	1.1–4.9	0.028 *
Throat specimen	3.1	1.4–6.9	0.005 **

Model performance: Nagelkerke R^2^ = 0.742, Hosmer–Lemeshow test *p* = 0.456 (good fit). Statistical significance: * *p* < 0.05, ** *p* < 0.01, *** *p* < 0.001.

**Table 5:** PCR Primers for Resistance Gene Detection.

Gene	Primer Sequence (5′ → 3′)	Product (bp)	Reference
*mef(A/E)*	F: CGT CAA AGA CAC GTG AAA AAC T R: CTT CTG TGT ACA TAA TTA ACC AGA	348	[26]
*msr(D)*	F: ACA AAA CTT TGG GAA ATG TTT GG R: GTT TGC AGC TTC TGA TTA TCG	482	[27]
*tet(K)*	F: GCT GAT GAT GGT CAA TGA C R: CTT GAC CAA AGA GGA GTT G	260	[27]

F = Forward primer; R = Reverse primer; bp = base pairs.

## Data Availability

Raw data supporting the conclusions of this study are available from the corresponding author upon reasonable request, subject to ethical approvals and institutional policies.

## Supplementary Materials

The following supporting information can be downloaded at: https://www.mdpi.com/article/10.3390/antibiotics14111148/s1, Table S1: Genome Accession Numbers for Regional Comparison; Table S2: Genome Accession Numbers for Regional Comparison; Table S3: The complete distribution of resistance and susceptibility patterns for all isolates, including erythromycin, clindamycin, and multidrug; Table S4: Distribution of resistance genes among UAE, India, and Saudi Arabia isolates; Table S5: Comparative analysis of antibiotic resistance across selected classes (Macrolides, Tetracyclines, and Lincosamides) among Streptococcus isolates from the UAE, India, and Saudi Arabia; Figure S1: Heatmap showing the prevalence of mef(A), msr(D), and tet(K) among Streptococcus isolates from the UAE, India, and Saudi Arabia; Figure S2: Heatmap illustrating the percentages of different antibiotic classes used in the UAE, India, and Saudi Arabia.
