# Supplementary material for: Efflux-Mediated Macrolide Resistance in Clinical Streptococcus Isolates: A Comparative Molecular Study
Source: Antibiotics (Basel). 2025 Nov 13;14(11):1148. doi: 10.3390/antibiotics14111148 (PMC12649515; doi:10.3390/antibiotics14111148)
Supplement: Supplementary file 1 [file antibiotics-14-01148-s001.zip › antibiotics-3897653-supplementary.pdf]

Supplementary Materials

Supplementary Table S1: Genome Accession Numbers for Regional Comparison

Saudi Arabia Isolates (n=15)

No.	WGS Accession	BioSample	SRA Run
1	DAYSUW000000000.1	SAMEA114733325	ERR12370235
2	DAYSUU000000000.1	SAMEA114733344	ERR12370254
3	DAYSUG000000000.1	SAMEA114733340	ERR12370250
4	DAYSUH000000000.1	SAMEA114733345	ERR12370255
5	DAYSUF000000000.1	SAMEA114733357	ERR12370267
6	DAYSUE000000000.1	SAMEA114733379	ERR12370289
7	DAYSDS000000000.1	SAMEA114733322	ERR12370232
8	DAYSTW000000000.1	SAMEA114733337	ERR12370247
9	DAYSTT000000000.1	SAMEA114733342	ERR12370252
10	DAYSTS000000000.1	SAMEA114733350	ERR12370260
11	DAYSTR000000000.1	SAMEA114733352	ERR12370262
12	DAYSTQ000000000.1	SAMEA114733355	ERR12370265
13	DAYSTP000000000.1	SAMEA114733363	ERR12370273
14	DAYSTN000000000.1	SAMEA114733374	ERR12370284
15	DAYSTK000000000.1	SAMEA114733389	ERR12370299

Supplementary Table S2: Genome Accession Numbers for Regional Comparison

India Isolates (n=15)

No.	Isolate ID	BioSample	Assembly
1	TVM3	SAMN07191007	GCA_002224185.1
2	B44415	SAMN07191006	GCA_002224195.1
3	3b00fc60-cfec-11e5-bc69-3c4a9275d6c6	SAMEA4038884	GCA_901292685.1
4	3b21f1e0-cfec-11e5-bc69-3c4a9275d6c6	SAMEA4035171	GCA_901292765.1
5	3c0266d0-cfec-11e5-bc69-3c4a9275d6c6	SAMEA4040383	GCA_901293145.1
6	3c299de0-cfec-11e5-bc69-3c4a9275d6c6	SAMEA4034884	GCA_901293215.1
7	3c349a60-cfec-11e5-bc69-3c4a9275d6c6	SAMEA4036445	GCA_901293285.1
8	3c67b850-cfec-11e5-bc69-3c4a9275d6c6	SAMEA4036483	GCA_901293405.1
9	3c872730-cfec-11e5-bc69-3c4a9275d6c6	SAMEA4034887	GCA_901293535.1
10	3ca3d6f0-cfec-11e5-bc69-3c4a9275d6c6	SAMEA4034888	GCA_901293695.1
11	3cd40eb0-cfec-11e5-bc69-3c4a9275d6c6	SAMEA4038885	GCA_901293815.1
12	3d3ede70-cfec-11e5-bc69-3c4a9275d6c6	SAMEA4035180	GCA_901294205.1
13	3d8b29b0-cfec-11e5-bc69-3c4a9275d6c6	SAMEA4034896	GCA_901294435.1
14	3d800620-cfec-11e5-bc69-3c4a9275d6c6	SAMEA4039831	GCA_901294505.1
15	3da170d0-cfec-11e5-bc69-3c4a9275d6c6	SAMEA4034897	GCA_901294525.1

Supplementary Table S3: The complete distribution of resistance and susceptibility patterns for all isolates, including erythromycin, clindamycin, and multidrug

**Resistance Pattern**	**Percentage (%)**
**Single antibiotic resistance**	
Erythromycin	39
Clindamycin	31
Penicillin	1
**Dual antibiotic resistance**	
Erythromycin + Clindamycin	22
Oxacillin + Levofloxacin	1
Teicoplanin + Erythromycin	1
**Multiple antibiotic resistance (≥3)**	
Erythromycin + Cotrimoxazole + Oxacillin	1
Oxacillin + Erythromycin + Clindamycin + Teicoplanin	2
Clindamycin + Erythromycin + Tetracycline + Oxacillin	1
Levofloxacin + Rifampin + Penicillin + Erythromycin + Teicoplanin + Ciprofloxacin + Clindamycin	1
**Susceptibility Patterns(senstivie)**	**Percentage (%)**
**Dual antibiotic Susceptibility**	
Penicillin + Vancomycin	48
**Multiple antibiotic Susceptibility (≥3)**	
Penicillin + Vancomycin + Clindamycin	43
Cotrimoxazole + Levofloxacin + Linezolid + Vancomycin	1
Cotrimoxazole + Erythromycin + Clindamycin + Vancomycin + Tetracycline	1
Clindamycin + Levofloxacin + Tetracycline + Vancomycin + Linezolid	1
Penicillin + Vancomycin + Erythromycin	1
Vancomycin + Penicillin + Cefaclor + Ampicillin	1
Erythromycin + Vancomycin + Levofloxacin + Linezolid + Teicoplanin + Cotrimoxazole + Clindamycin	2
Cotrimoxazole + Levofloxacin + Vancomycin + Linezolid	1
None	1

Supplementary Table S4 : Distribution of resistance genes among UAE, India, and Saudi Arabia isolates

Gene	UAE (n=100)	India (n=15)	Saudi Arabia (n=15)	p-value*
mef(A/E)	31 (31.0%)	13 (86.7%)	0 (0.0%)	<0.001 ***
msr(D)	3 (3.0%)	13 (86.7%)	0 (0.0%)	<0.001***
tet(K)	0 (0.0%)	0 (0.0%)	0 (0.0%)	1.000 (NS)
erm(B)	0(0.0%)	0(0.0%)	3(20.0%)	0.0013**

Fisher's exact test with Bonferroni correction (α=0.0125)Statistical significance is denoted as follows: *p < 0.05, **p < 0.01, *** **p < 0.001, 1.000 is not significant**

Supplementary Table S5: Comparative analysis of antibiotic resistance across selected classes (Macrolides, Tetracyclines, and Lincosamides) among Streptococcus isolates from the UAE, India, and Saudi Arabia

Antibiotic Class	Overall Chi-square p-value	Fisher's Exact Test UAE vs India	Fisher's Exact Test UAE vs Saudi	Fisher's Exact Test India vs Saudi
Macrolides	0.243 (NS)	0.224 (NS)	0.548 (NS)	1.000 (NS)
Tetracyclines	p < 0.001 ***	p < 0.001 ***	0.0001 ***	1.000 (NS)
Lincosamides	0.0001 ***	0.0004 ***	0.012 **	0.598 (NS)

Overall comparisons were performed using Chi-square tests; pairwise comparisons were assessed using Fisher's exact test. Significance levels: *p < 0.05, **p < 0.01, *****p < 0.001; NS = not significant.**

Supplementary Figure S1:

Supplementary Figure S1:: Heatmap showing the prevalence of mef(A), msr(D), and tet(K) among Streptococcus isolates from the UAE, India, and Saudi Arabia

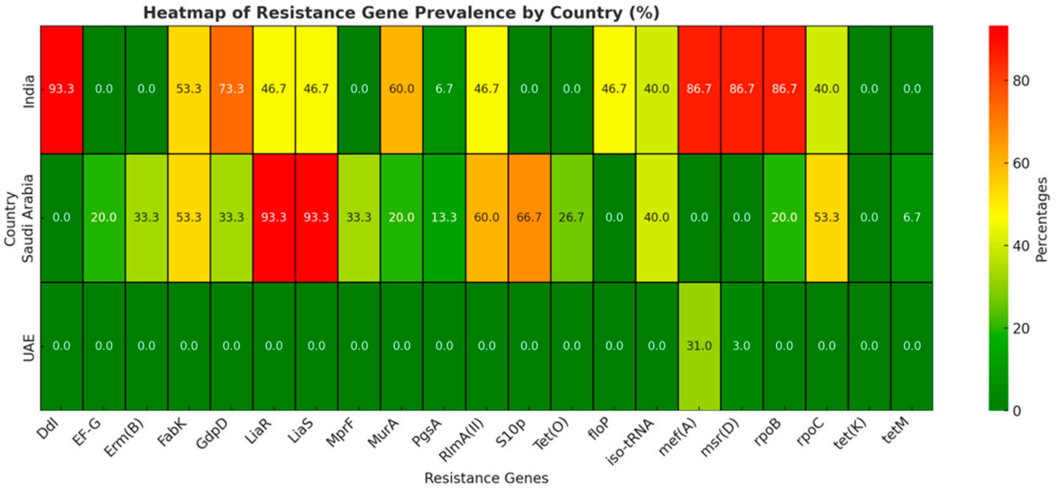

Heatmap illustrating the prevalence of resistance genes among Streptococcus isolates from the UAE (n = 100), India (n = 15), and Saudi Arabia (n = 15). Color intensity indicates prevalence (%) depending on thresholds, while values inside the cells indicate percentages. Only mef(A) was commonly found (31.0%) in the UAE isolates (n=100), all other genes were absent (0%), and msr(D) was found at a lesser rate (3%). In contrast, Indian isolates (n=15) exhibited markedly higher prevalence of several genes, particularly *msr(D)* (86.7%) and *mef(A)* (86.7%), whereas *tet(K)* was absent (0%). Saudi Arabian isolates (n=15) demonstrated the highest prevalence of *LiaR* (93.3%), *LiaS* (93.3%), and *mprF* (33.3%), while *mef(A)*, *msr(D)*, and *tet(K)* were all absent.

Supplementary Figure S2: Heatmap illustrating the percentages of different antibiotic classes used in the UAE, India, and Saudi Arabia

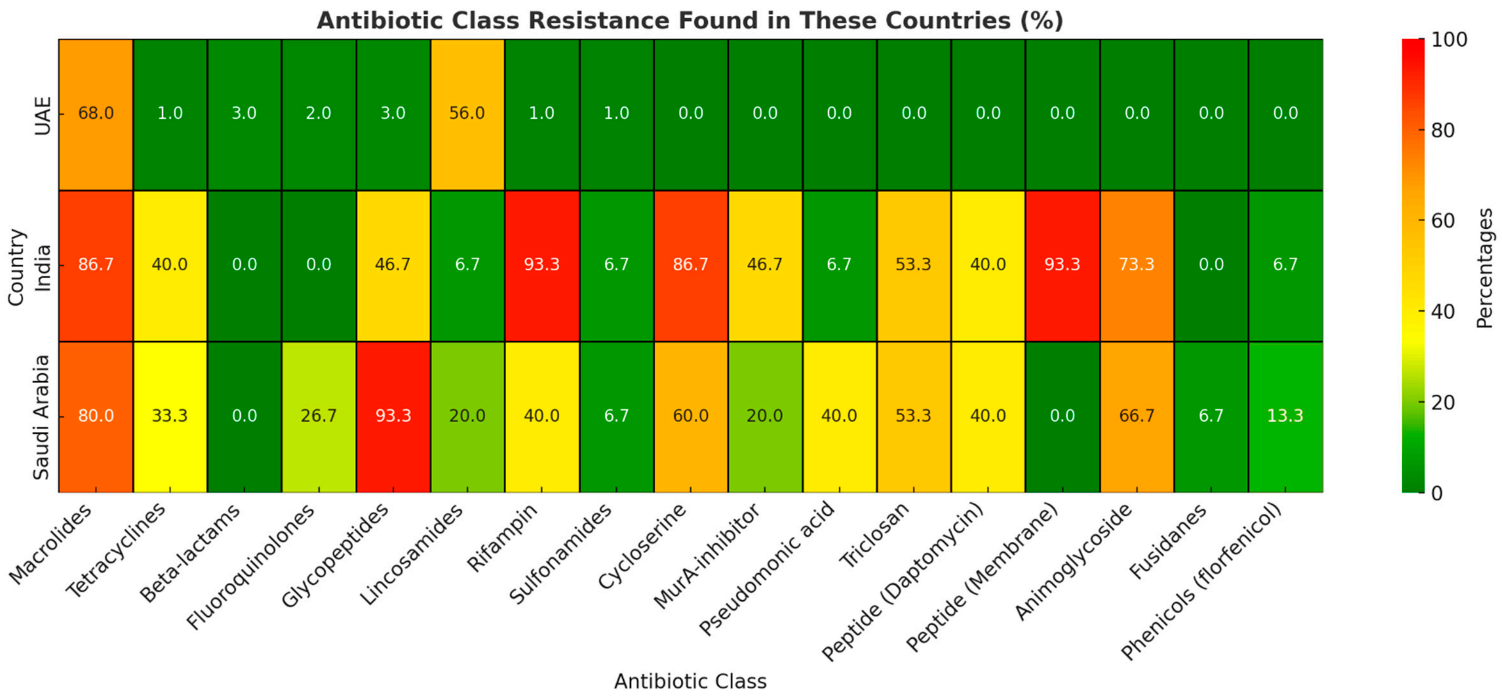

Heatmap illustrating the percentages of different antibiotic classes used in the UAE (n = 100), India (n = 15), and Saudi Arabia (n = 15). Resistance values are represented both numerically within each cell and by a color gradient (green = low resistance, red = high resistance). The scale bar on the right indicates resistance percentages (0–100%). Across all three countries, macrolide resistance—linked to *mef(A)/msr(D)*—was the highest, reaching 86.7% in India, 80% in Saudi Arabia, and 68% in the UAE. In contrast, tetracycline was the lowest, with rates of 1% in the UAE, 33.3% in Saudi Arabia, and 40% in India.

Raw data (LINK)
